# Age‐related arterial stiffness and cerebrovascular dysfunction in mice correlate with cognitive impairment but are not reduced with long‐term ALT‐711 treatment

**DOI:** 10.14814/phy2.70982

**Published:** 2026-06-17

**Authors:** Emily H. Reeve, Abigail E. Cullen, Skylyn J. Ferguson, Maxwell Braker, Ainsley P. Hogan, Alexandra Famiano, Ashley E. Walker

**Affiliations:** ^1^ Department of Human Physiology University of Oregon Eugene Oregon USA

**Keywords:** aging, Alagebrium chloride, arterial stiffness, collagen crosslinking, endothelial dysfunction

## Abstract

Age‐related increases in large artery stiffness contribute to cerebrovascular dysfunction and cognitive impairment. Alagebrium Chloride (ALT‐711) is a collagen crosslink breaker that reduces vascular stiffness. We hypothesized that long‐term treatment with ALT‐711 would reverse age‐related large artery stiffness, thereby preserving cerebral artery function and cognitive function. We treated old male and female C57BL/6 mice (20 months) with ALT‐711 (1 mg/kg/day) or vehicle via oral gavage for 4 months and measured arterial stiffness, cerebral artery endothelial function, cognitive function, and collagen crosslinking. Treatment with ALT‐711 did not significantly reduce stiffness in large arteries or cerebral arteries. There was no effect of ALT‐711 on cerebral artery endothelial function, cognitive function, or collagen crosslinking. However, we found that collagen crosslinking was greater in old mice than in young, untreated C57BL/6 mice. In old mice, aortic collagen crosslinking was correlated with carotid artery passive stiffness. Additionally, among old mice, the passive stiffness of the cerebral artery negatively correlated with cerebral artery endothelial function and cognitive function. Cerebral artery endothelial function was negatively correlated with Frailty Index. In sum, age‐related cerebral artery stiffness is negatively correlated with cognitive function and may be a promising therapeutic target to combat cerebrovascular dysfunction and cognitive decline with age.

## 1 INTRODUCTION

Stiffening of large arteries is an age‐related change in the vascular system that increases the risk of neurodegeneration (Chirinos et al., 2019; Cooper & Mitchell, 2016). Stiff arteries lose the ability to damp pulse pressure. This increases pulse pressure transmitted to smaller resistance vessels. (van Sloten et al., 2015). Increased mechanical and circumferential stress from high pulse pressure damages the fragile endothelial layer that is crucial for vascular function (Rabkin, 2012; Thorin‐Trescases et al., 2018). Cerebrovascular dysfunction, including reductions in cerebral blood flow, is one of the greatest predictors of Alzheimer's disease development and is a consequence of increases in large artery stiffness (Mitchell et al., 2004; Ruitenberg et al., 2005; Stone et al., 2015).

Previous work by our lab indicates that elevated large‐artery stiffness impairs cerebral artery endothelium‐dependent dilation (EDD) by reducing nitric oxide (NO)‐mediated vasodilation (Walker et al., 2015). Recently, we showed that pyridoxamine, a vitamin B6 isomer known to reduce the formation of advanced glycation end products (AGEs), attenuates large artery stiffness and preserves cerebrovascular EDD, with modest effects on cognition (Reeve et al., 2023). However, pyridoxamine may act through non‐AGE‐related mechanisms, as it functions as an antioxidant and affects multiple target organs.

Systemic and exogenous AGE levels rise with biological age, but interventions specifically designed to prevent this formation have low success rates (Luevano‐Contreras & Chapman‐Novakofski, 2010; Peppa et al., 2008; Semba et al., 2009). Rather than preventing the formation of AGEs, another strategy is to reverse the collagen crosslinking induced by these AGEs (Vasan et al., 1996). AGEs, such as pentosidine, form a collagen crosslink bridge within the extracellular matrix of the vascular wall, increasing the structural stiffness of multiple tissue types (Mammoto et al., 2022). These collagen crosslinks form in the large arteries, contributing to the age‐related increases in stiffness observed in these structures (Sajithlal et al., 1998). Alagebrium Chloride (ALT‐711) is a compound that breaks these AGE‐induced collagen crosslinks. ALT‐711 has been shown to decrease arterial stiffness and to improve large artery vascular function and left ventricular function in rodents, primates, and humans (Kass et al., 2001; Susic et al., 2004; Vaitkevicius et al., 2001; Wolffenbuttel et al., 1998). However, the effects of ALT‐711 on cognitive and cerebrovascular function have not yet been investigated.

Thus, the purpose of this study was to investigate the effects of the collagen‐crosslink breaker ALT‐711 on preserving cerebrovascular and cognitive function by reducing large artery stiffness. We hypothesized that old mice treated with ALT‐711 would exhibit reduced large artery stiffness and preserved cerebrovascular and cognitive function compared with control mice. To test these hypotheses, we treated old C57BL/6 mice with ALT‐711 for 16 weeks and measured aortic stiffness monthly. At the end of the intervention, we assessed frailty, cognitive function, cerebral endothelial function, and passive stiffness of the cerebral and carotid arteries. We also measured aortic hydroxyproline content to compare collagen crosslinking in control and ALT‐711 treated animals with that of young physiological controls. Lastly, we pooled all data and ran a Pearson correlation to determine relationships among large artery function, small artery function, collagen crosslinking, frailty, and cognition.

## 2 METHODS

### 2.1 Intervention

Aged C57BL/6 male and female mice were obtained from the National Institute on Aging colony at Charles River (20 months, *n* = 16). Animals were allowed a 2‐week acclimation period in our research facility before intervention and baseline testing began. All mice were fed a normal chow diet (Lab Diet, PicoLab Rodent Diet 20, 5053) with ad libitum food and water and were housed in an animal care facility on a 12/12‐h light–dark cycle at 24°C. Animals were matched for baseline aortic stiffness values and assigned to the treatment or control group by block randomization (*n* = 8/group; 4 males, 4 females). Mice received daily oral gavage of either ALT‐711 (1 mg/kg/day, TCI America, cat #A3166) or vehicle (distilled water) for 3 weeks, followed by one recovery week, for a total of 16 weeks. A young C57BL/6 control group was used for comparison for the hydroxyproline assay (6 months, *n* = 7, all males). Mice were euthanized by exsanguination under isoflurane immediately before ex vivo vascular studies. After euthanasia, the heart, liver, spleen, soleus, gastrocnemius (gastroc), and white adipose tissue (WAT) were collected, and their wet weights were recorded. All animal procedures conform to the *Guide to the Care and Use of Laboratory Animals* (8th edition, revised 2011) and were approved by the Institutional Animal Care and Use Committees at the University of Oregon. All data reported adhere to the ARRIVE guidelines for conducting animal research.

### 2.2 Frailty index

To measure biological aging parameters, we used a frailty index test. Mouse frailty was assessed using a previously studied 31‐item FI based on established clinical signs of deterioration in C57BL/6J mice, as previously described (Cole et al., 2022; Whitehead et al., 2014). Vision loss was excluded because it is difficult to measure accurately in mice. Frailty assessment included evaluation of the integumentary, musculoskeletal, vestibulocochlear/auditory, ocular, nasal, digestive, urogenital, and respiratory systems, as well as signs of discomfort, body weight, and body surface temperature. The severity of each deficit was assessed and assigned a score of 0, 0.5, or 1, with higher scores indicating more severe frailty. Each mouse was examined at approximately the same time of day, following the same order of assessments, within 7 days of their planned vascular reactivity study.

### 2.3 Pulse wave velocity (PWV)

Mice were anesthetized by 2% inhaled isoflurane in 100% oxygen and situated on a temperature‐controlled heating pad (37°C) to maintain body temperature. Electrodes were placed on the distal parts of limbs to record ECG information, while 20 MHz Doppler transducers were placed on the aortic arch and abdominal aorta. We measured pulse wave velocity (PWV) using the Indus Doppler Flow Velocity System (Webster, Texas) from the descending aorta to the abdominal aorta (Donato et al., 2013; Henson et al., 2014). Three consecutive measurements at each time point (5 total; baseline and months 1–4) were analyzed by the researchers, with inter‐researcher variability <10%. PWV can be significantly affected by the distance and location of the probes on the animals during measurement collection. Thus, to control for this, the location of the probes was tracked for each mouse, and distances were kept within ±1 mm of the individual baseline measurement.

### 2.4 Passive stiffness

Carotid arteries and posterior cerebral arteries (PCAs) from each mouse were isolated and mounted on a pressure myograph (DMT Inc., Denmark) as previously described (Lesniewski et al., 2009; Walker et al., 2015). Passive stiffness measurements on the PCA were performed after ex vivo pressure myography (see methods below). Arteries were pressurized and incubated in a calcium‐free physiological salt solution to eliminate the effects of myogenic tone.

We recorded changes in lumen diameter and medial wall thickness following increases in intraluminal pressure from 5 to 85 cmH2O (3.7–62.5 mmHg) in 5 cmH_2_O increments (Walker et al., 2015). Stress was calculated as follows:

σ=PD/2WT

where *P* is the pressure in dyne cm^−2^, *D* is the lumen diameter, and WT is the wall thickness.

Strain was calculated as follows:

$$\varepsilon =\left(D-\mathrm{Di}\right)/\mathrm{Di}$$

where *D* is the diameter, and *D*i is the initial starting diameter. Data for each artery were fit to the curve:

$$\sigma =\mathrm{\sigma i}e^{\beta \varepsilon }$$

where σi is the initial starting stress (5 cmH_2_O), and *β* is the slope of the tangential elastic modulus versus stress. A higher *β* represents a stiffer artery. From this slope, we also calculated Young's elastic modulus (low and high) (Baumbach & Hajdu, 1993; Dunn & Gardiner, 1997; Izzard et al., 2006).

### 2.5 Cerebral artery endothelial function

To assess endothelial function in cerebral arteries, we used ex vivo pressure myography. PCAs from each mouse were excised and placed in a myograph chamber (DMT Inc., Aarhus, Denmark) with physiological salt solution that contained 145.0 mmNaCl, 4.7 mmKCl, 2.0 mm CaCl_2_, 1.17 mm MgSO_4_, 1.2 mm NaH_2_PO_4_, 5.0 mm glucose, 2.0 mm pyruate, 0.02 mm EDTA, 3.0 mm MOPS buffer and 1 g/100 mL BSA, pH 7.4 at 37°C, cannulated onto glass micropipettes and secured with nylon (11–0) suture. All arteries were pre‐constricted with phenylephrine (PE, 10–60 μM to obtain 15%–40% pre‐constriction, Sigma, cat #1533002), and we measured changes in luminal diameter in response to increasing concentrations of the endothelium‐dependent dilator acetylcholine (ACh, 1 × 10^–9^ to 1 × 10^–4^ M, Sigma, cat #1008501). Additionally, we verified that constriction to PE held above 15% throughout the first dose of ACh. If arteries did not adequately constrict, the dose response was excluded from analysis. ACh dose responses were repeated after incubation with the nitric oxide synthase (NOS) inhibitor, *N*ω‐Nitro‐L‐arginine methyl ester hydrochloride (L‐NAME, 0.1 mM, 30‐min incubation, Sigma, cat #N5751). We also measured changes in diameter in response to increasing doses of the endothelium‐independent dilator, sodium nitroprusside (SNP, 1 × 10^−10^ to 1 × 10^−4^ M, Sigma, cat #1614501).

### 2.6 Cognitive testing

We assessed cognitive function by measuring instinctual nest building behavior (Deacon, 2012). Mice were individually housed overnight in a cage with food, water, and a condensed cotton nestlet. In the morning, the nests were scored on a scale of 1–5 for quality.

The Morris Water Maze cognitive test was used to assess learning and spatial memory using a protocol previously described (Duvoisin et al., 2010). Mice underwent a 3‐day training period, with 2 sessions per day and 3 trials per session. On the fourth day, a probe trial was performed, followed by a beacon trial. All sessions were recorded and analyzed with EthoVision XT12 software (Noldus, Wageningen, the Netherlands).

Motor coordination testing was performed using a rotarod (47,650 Rota‐Rod NG, Ugo Basile, Gemonio, Italy) over 2 days. The first day was a training session, and mice were required to remain on the rod while it rotated at 4 rpm for 90 s. The second day consisted of three trials, with a 10‐min rest between trials, during which the rod accelerated from 4 to 40 rpm, with a cutoff time of 5 min. The time was recorded for when the mouse fell from the rod or rotated around the rod two consecutive times (Deacon, 2013).

### 2.7 Collagen crosslinking

To estimate the collagen concentration in tissue, we used a hydroxyproline assay modified for aortic tissue (Faakye et al., n.d.; Abbott et al., 2021; Smith et al., 2016). Aortas were thawed and digested in a pepsin solution (Thermofisher, cat #AAJ6167906) to separate the soluble and insoluble collagen protein. The aorta samples and hydroxyproline standard dilutions were hydrolyzed by incubation for 24 h at 110°C in 6 M HCl. Each sample was mixed with 150 μL isopropanol and 75 μL chloramine‐T (Millipore Sigma, cat #23270, St. Louis, MO, USA) in citrate buffer, then oxidized for 10 min at room temperature. The samples were then mixed with 1 mL of a 3:13 solution of Ehrlich reagent: isopropanol and incubated for 30 min at 58°C. The Ehrlich reagent consisted of *p*‐ dimethylaminobenzaldehyde (Sigma‐Aldrich, cat #156477, St. Louis, MO, USA), 100% ethanol, and sulfuric acid. The reaction was stopped on ice and quantified by measuring absorbance at 558 nm in duplicate with a spectrophotometer (BioTek Instruments, Inc., Winooski, VT). Hydroxyproline concentration (μg/ml/mg of tissue) was then determined using a standard curve of trans‐4‐hydroxy‐l‐proline (Sigma‐Aldrich, cat #H5534, St. Louis, MO, USA). For this assay, we used 2 physiological replicates per sample and 3 technical replicates per standard. From our samples, we determined the hydroxyproline concentration of a pepsin insoluble fraction (PIF), pepsin soluble fraction (PSF), and total hydroxyproline content (PIF + PSF). PIF was used as a measurement of crosslinked collagen, while PSF measured non‐crosslinked collagen.

### 2.8 Statistical analysis

All data were tested for normality using a Shapiro–Wilk test. For comparisons of non‐normally distributed data, appropriate nonparametric tests were used. A Student's *t*‐test was used to compare average heart rate data. For PWV measurements, we used a Mann–Whitney test to compare between‐group PWV (unpaired) at each time point. Baseline and Month 4 *p*‐values were corrected for multiple comparisons. Since baseline and month 4 data were normally distributed, we also used a 2‐way RM‐ANOVA to compare treatment, time, and interaction effects. For β‐stiffness and elastic modulus (high and low) measurements in the carotid, a Mann–Whitney test was used to compare the ALT‐711 and control groups. An unpaired Student's *t‐*test was used for comparison of lumen diameter and wall thickness. For all PCA passive stiffness data, a Student's *t*‐test was used to compare between treatment groups. For pressure myography dose‐responses, we normalized the data by transforming it to the ln (% maximal dilation). We then performed a 3‐way RM ANOVA comparing the entire dose response across treatment groups and bath condition (ACh vs. ACh + L‐NAME). For SNP dose responses, we analyzed the data using a mixed‐effects model due to missing values after transformation. For hydroxyproline measurements, a one‐way ANOVA was used to compare values between a young, ALT‐711, and control groups.

Pearson correlations were performed across all old mice (control and ALT‐711 combined) to assess relationships among vascular stiffness, endothelial function, and collagen crosslinking. Correlation data were all normally distributed and calculated using SPSS 26. All other comparisons were run in GraphPad Prism 10. A *p*‐value <0.05 was considered statistically significant. For Bonferroni corrections, a *p*‐value <0.025 was considered statistically significant. Data are displayed as Mean ± standard deviation (SD).

## 3 RESULTS

### 3.1 Animal characteristics

A detailed display of animal age, tissue weights, and frailty index is provided in Table S1.

### 3.2 Large artery stiffness

**FIGURE 1:**
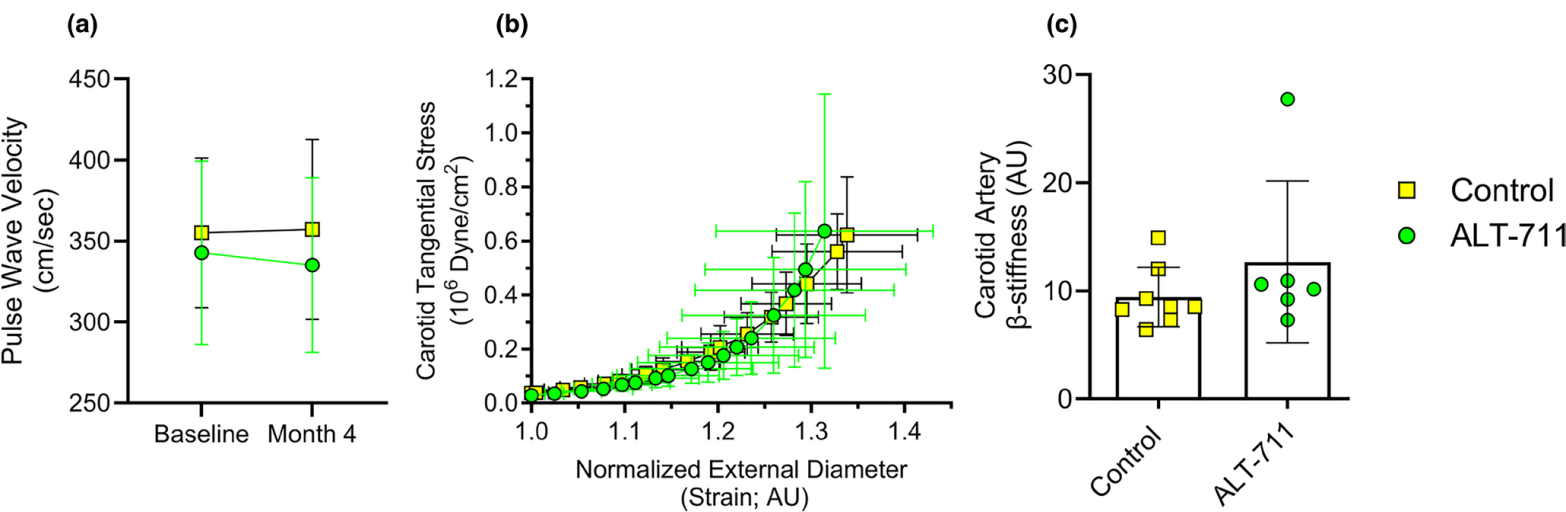
ALT‐711 treatment did not reverse large artery stiffness in aged mice. (a) Pulse Wave Velocity (PWV) in control and ALT‐711 treated mice from baseline to month 4 of intervention, *N* = 7–8/group. (b) Stress–strain curve from carotid artery passive stiffness measurements in control and ALT‐711 treated mice, *N* = 7–8/group. (c) Carotid artery β‐stiffness in control and ALT‐711 treated mice, *N* = 7–8/group. Data are Mean ± SD. Statistics include a Mann–Whitney and 2‐way RM‐ANOVA (a) and a Mann–Whitney (c).

**FIGURE 2:**
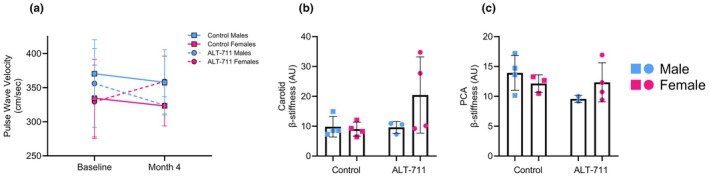
Large artery stiffness outcomes in treatment groups separated by sex. (a) Pulse Wave Velocity (PWV) in control and ALT‐711 treated mice from baseline to month 4 of intervention. *N* = 3–4/group. (b) Stress–strain curve from carotid artery passive stiffness measurements in control and ALT‐711 treated mice, *N* = 3–4/group. (c) Carotid artery β‐stiffness in control and ALT‐711 treated mice, *N* = 3–4/group. Males are indicated in blue and females are indicated in pink. Data are Mean ± SD.

Aortic PWV did not differ between control mice and mice treated with ALT‐711 at the same time point during the intervention (*p* > 0.05 for all; Figure S1). Additionally, there were no differences in aortic PWV between treatments, time (baseline vs. month 4), or their interaction (*p* > 0.05 for all, Figure 1a). Average heart rate each month did not significantly differ between the treatment and control groups (*p* > 0.05, Table S2). We calculated β‐stiffness for the carotid artery using a stress–strain curve (Figure 1b). There was no significant difference in carotid artery β‐stiffness between the ALT‐711 and control groups (*p* > 0.05, Figure 1c). There were no differences in elastic modulus at low and high pressures, lumen diameter, and wall thickness between groups in the carotid artery (Table 1). Data were separated by sex to assess whether sex differences exist in arterial stiffness outcomes with ALT‐711 treatment (Figure 2). Due to the low sample size, statistical analysis of these data could not be performed. However, there does not appear to be a difference in the overall response to ALT‐711 for arterial stiffness outcomes between sexes. Overall, the PWV and passive stiffness data suggest that ALT‐711 treatment did not prevent or reverse large artery stiffness in these mice.

**TABLE 1:** Carotid and PCA passive response values.

Variable	Group		*p*‐value
	Control	ALT‐711	
*N*	8	7	
Carotid EM_low_	0.63 ± 0.2	0.92 ± 0.5	0.10
Carotid EM_high_	2.91 ± 2.6	2.19 ± 3.2	0.27
*N*	7	7	
PCA EM_low_	0.98 ± 0.4	1.14 ± 0.4	0.23
PCA Em_high_	4.39 ± 2.8	3.05 ± 2.9	0.19
*N*	8	6	
Carotid lumen diameter (μm)	396 ± 37	400 ± 41	0.40
Carotid wall thickness (μm)	37 ± 9	38 ± 12	0.44
*N*	7	7	
PCA lumen diameter (μm)	141 ± 13	149 ± 14	0.14
PCA wall thickness (μm)	16 ± 4	15 ± 2	0.36

*Note*: ALT‐711 treatment does not affect passive stiffness, diameter, or thickness of the carotid artery and PCA. Values for elastic modulus (EM) at low and high pressures calculated from stress/strain curves. Values for lumen diameter and wall thickness are from passive pressure myography at 51 mmHg. Data are Mean ± SD. Statistics include a Mann‐Whitney for carotid arteries and a one‐tailed Student's *t*‐test for PCAs. Posterior cerebral artery (PCA).

### 3.3 Cerebrovascular endothelial function

**FIGURE 3:**
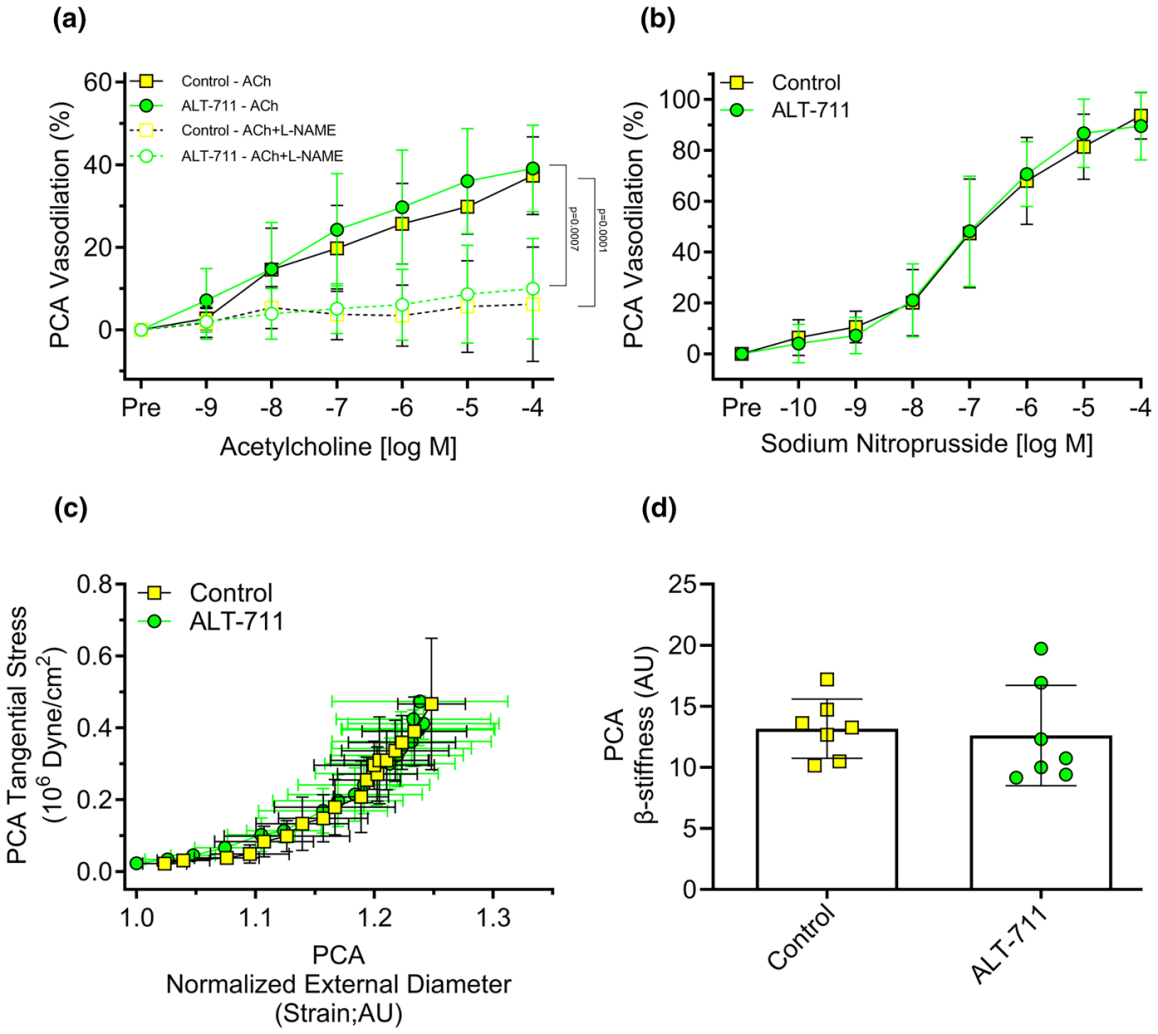
ALT‐711 treatment did not affect cerebrovascular function and stiffness. (a) Dose response curves to ACh and ACh + L‐NAME for control and ALT‐711 treated mice, *N* = 8/group. (b) Dose response curves to SNP for control and ALT‐711 treated mice, *N* = 8/group. (c) Stress–strain curves from PCA passive stiffness measurements in control and ALT‐711 treated mice, *N* = 7/group. (d) PCA β‐ stiffness in control and ALT‐711 treated mice, *N* = 7/group. Data are Mean ± SD. Statistics include an unpaired *t*‐test with (a) and without (b) Bonferroni correction, and a Mann–Whitney test (c).

We found no difference in the dose response or percent maximal PCA vasodilation to ACh between the control and treatment groups (dose response: *p* = 0.87, maximal: *p* = 0.30, Figure 3a). There was a main effect for a reduction in the response to ACh after the application of L‐NAME (dose response: *p* < 0.0001, maximal: *p* < 0.001). However, there was no significant interaction between treatment group (ALT‐711 vs. control) and bath condition (ACh vs. ACh + L‐NAME), suggesting a similar response to L‐NAME between groups (dose response: *p* = 0.66; maximal: *p* = 0.32). This indicates that the endothelial NO signaling pathway contributed to ACh‐induced vasodilation, but ALT‐711 treatment did not hinder or enhance this pathway in cerebral arteries.

Lastly, the percent maximal vasodilation to SNP did not differ between treatment groups (*p* = 0.26, Figure 3b). The overall dose response to SNP was also not significantly different among treatment groups, indicating that smooth muscle cell response to NO was not different in ALT‐711 mice compared to control mice (*p* = 0.20).

### 3.4 Cerebral artery stiffness

We calculated PCA β‐stiffness using the stress–strain curve (Figure 3c). In the PCA, there was no difference in β‐stiffness between ALT‐711 treated mice and control mice (*p* > 0.05, Figure 3d). There were no differences in elastic modulus at low or high pressures, lumen diameter, or wall thickness between treatment groups (*p* > 0.05 for both, Table 1). These results suggest that ALT‐711 does not alter cerebral artery stiffness.

### 3.5 Collagen crosslinking

**FIGURE 4:**
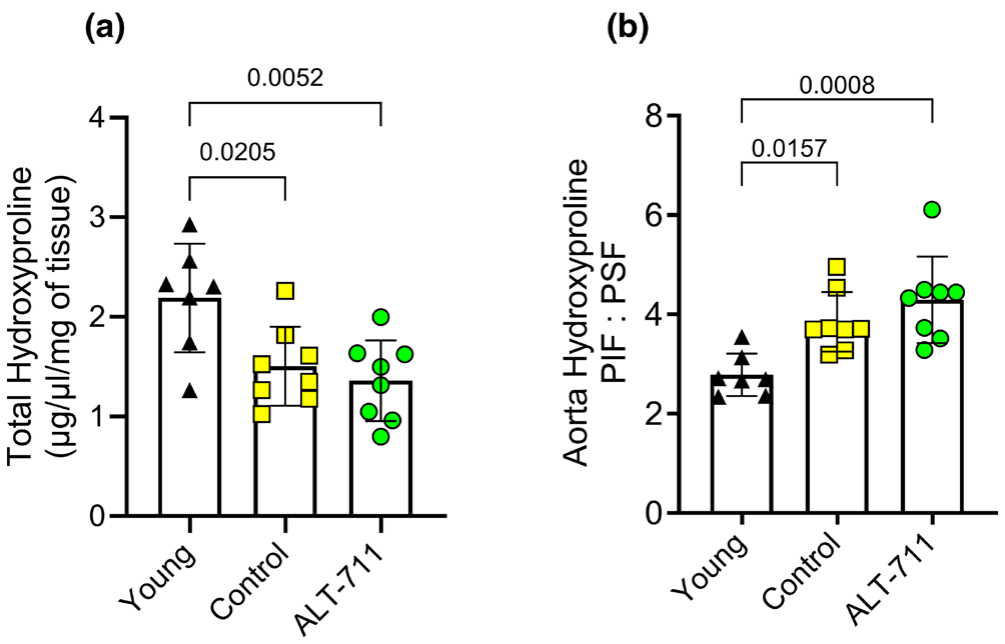
Aortic collagen crosslinking is greater with age but is not affected by ALT‐711 treatment. (a) Aorta total hydroxyproline content, a measure of collagen, in young (6–7 months), control, and ALT‐711 treated mice, *N* = 7–8/group. (b) Aorta Hydroxyproline PIF: PSF ratio from hydroxyproline assay, an estimate of crosslinked: non‐crosslinked collagen, in aortas of young, control, and ALT‐711 treated mice, *N* = 7–8/group. Pepsin soluble fraction (PSF), pepsin insoluble fraction (PIF). Data are Mean ± SD. Statistics include a one‐way ANOVA and Tukey test for multiple comparisons.

Total aortic hydroxyproline content was greater in young mice compared to both old control (*p* = 0.02) and old ALT‐711‐treated mice (*p* = 0.005, Figure 4a). Both ALT‐711‐treated mice (*p* < 0.001) and control mice (*p* < 0.01) had a greater PIF: PSF ratio, a measure of crosslinked: non‐crosslinked collagen, compared to young mice (Figure 4b).

### 3.6 Cognitive tests

**FIGURE 5:**
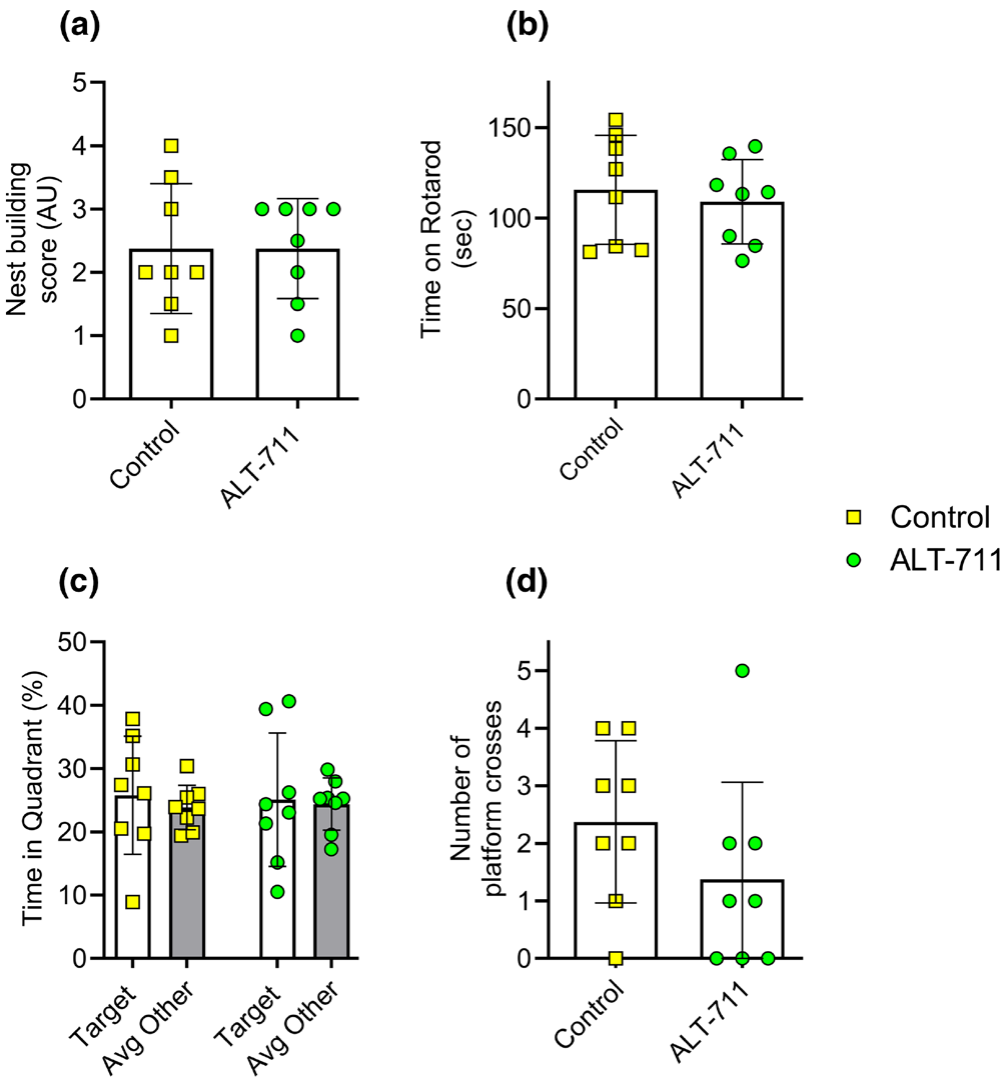
Long‐term treatment with ALT‐711 does not affect cognitive function. (a) Post‐treatment nest building scores in control and ALT‐711 treated mice, *N* = 8/group. (b) Average time spent on Rotarod in control and ALT‐711 treated mice, *N* = 8/group. (c) Time spent in the target quadrant and average of other 3 quadrants during the Morris water maze probe trial in control and ALT‐711 treated mice, *N* = 8/group. (d) Number of platform crosses during the Morris water maze probe trial in control and ALT‐711 treated mice, *N* = 8/group. Data are Mean ± SD. Statistics include a Student's *t*‐test (a, b, d) and 2‐way ANOVA (c).

There were no differences in post‐treatment nest building scores or the average time spent on the rotarod between control and ALT‐711‐treated animals post‐intervention (*p* > 0.05 for both, Figure 5a,b). For the Morris water maze test, there was no difference in the average time spent in the target quadrant during the probe trial between control and ALT‐711‐treated animals (*p* > 0.05, Figure 5c). Lastly, there was no difference in the number of times mice crossed the platform between control and ALT‐711‐treated mice (*p* > 0.05, Figure 5d).

### 3.7 Correlations

**FIGURE 6:**
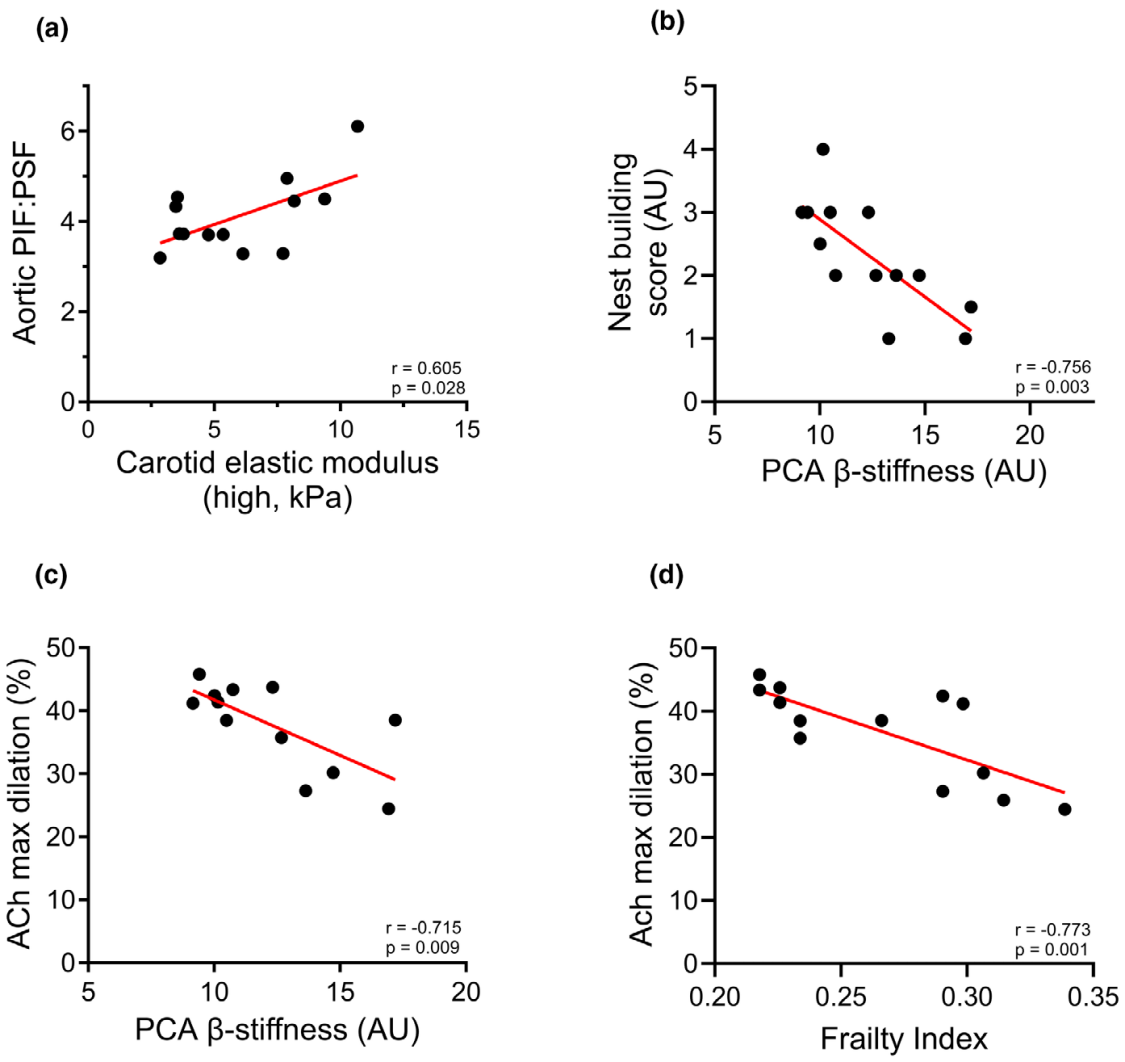
Cerebral artery stiffness is negatively correlated with cerebral artery endothelial function and cognition. Pearson's Correlations between (a) Carotid elastic modulus at high pressures and aortic hydroxyproline PIF:PSF. (b) PCA β‐stiffness and nest building score. (c) PCA β‐stiffness and PCA percent maximal vasodilation to ACh. (d) Frailty index and PCA percent maximal vasodilation to ACh. *N* = 12–14. The solid red line indicates the mean trendline. Statistics include a Pearson correlation coefficient computed in SPSS 30.0.

Among old mice, we found that the aortic PIF:PSF was positively correlated with carotid elastic modulus at high pressures (*r* = 0.605, *p* = 0.028, Figure 6a), indicating a link between collagen crosslinking and passive stiffness. PCA β‐stiffness was negatively correlated with nest building scores (*r* = −0.756, *p* = 0.003, Figure 6b) and PCA maximal vasodilation to ACh (*r* = −0.715, *p* = 0.009, Figure 6c), suggesting that passive cerebral artery stiffness relates to cognitive function and endothelial function. Lastly, maximal vasodilation to ACh in the PCA was negatively correlated with the Frailty Index (*r* = −0.773, *p* = 0.001; Figure 6d), indicating that cerebral artery endothelial function is associated with a marker of biological age.

**FIGURE 7:**
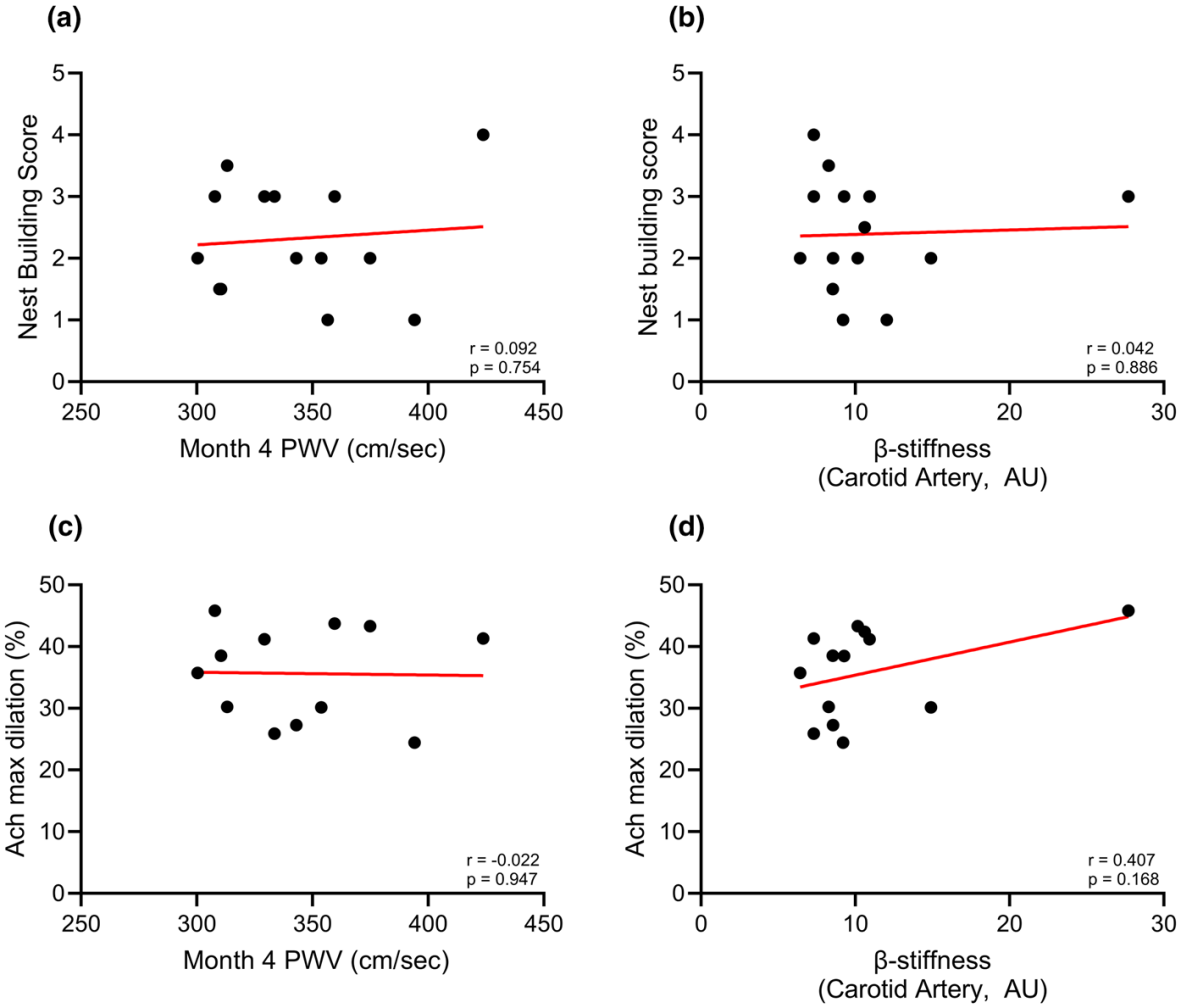
Large artery stiffness does not correlate with cerebral artery function and cognitive function. Pearson's Correlations between (a) Month 4 PWV and nest building score. (b) Carotid artery β‐stiffness and nest‐building score. (c) Month 4 PWV and PCA percent maximal vasodilation to ACh. (d) Carotid artery β‐stiffness and PCA percent maximal vasodilation to ACh. *N* = 12–14. The solid red line indicates the mean trendline. Statistics include a Pearson correlation coefficient computed in SPSS 30.0.

Additional correlations among large artery stiffness, cerebral artery function, and cognitive function were of interest to us but showed no significant relationships in this study. Nest building was correlated with neither large artery stiffness outcomes, such as Month 4 PWV (*r* = 0.092, *p* = 0.754, Figure 7a), nor carotid artery β‐stiffness (*r* = 0.042, *p* = 0.886, Figure 7b). Additionally, we found that PCA maximal vasodilation to ACh was correlated with neither Month 4 PWV (*r* = −0.022, *p* = 0.947, Figure 7c), nor carotid artery β‐stiffness (*r* = 0.407, *p* = 0.168, Figure 7d).

## 4 DISCUSSION

The results of our study suggest that long‐term ALT‐711 treatment in mice did not affect large artery stiffness, cerebral artery function, or cognitive impairment. However, we did find a few notable correlations of interest among the old mice. We found that large artery stiffness was associated with collagen crosslinking, as expected (Hayashi & Hirayama, 2017; Sell & Monnier, 2012). We also report on a novel finding that cerebral artery stiffness is associated with endothelial dysfunction and cognitive impairment. Lastly, we confirmed that the frailty index correlated with cerebral artery endothelial dysfunction, as previously reported from our findings in middle cerebral arteries from old, female mice (Cole et al., 2022).

### 4.1 Large artery stiffness and collagen crosslinking

Despite prior evidence indicating the beneficial effects of ALT‐711 on cardiovascular function, we saw no changes in the cardiovascular outcomes of aged rodents treated long‐term with the compound (Sell & Monnier, 2012; Susic et al., 2004; Toprak et al., 2017; Vaitkevicius et al., 2001). Previous studies demonstrated that ALT‐711 reduces large artery stiffness and improves vascular health in rodents (Freidja et al., 2012; Vasan et al., 1996; Wolffenbuttel et al., 1998). However, previous studies that found beneficial effects of ALT‐711 treatment in rodents used diseased models such as obesity, diabetes, or spontaneous hypertension, and often used short‐term treatment protocols of 1–3 weeks (Freidja et al., 2012; Susic et al., 2004; Wang et al., 2015). We adapted our long‐term treatment protocol based on a study by Susic et al., who found that the optimal dose of ALT‐711 to observe beneficial vascular effects in rats was 1 mg/kg/day for 4 months (Susic et al., 2004). Our findings of no effects from ALT‐711 could be due to our study of healthy, aging animals. Animals housed in our facility were free of pathogens and maintained under optimal living conditions, with a diet optimized to support their health.

Further work by Kass et al. (2001) with ALT‐711 in humans suggested that it improved arterial compliance and reduced pulse pressure in older adults with vascular stiffening (Kass et al., 2001). In contrast, one study by Hartog et al. that tested ALT‐711 treatment in patients with chronic heart failure showed no beneficial effects on cardiac function (Hartog et al., 2011). In our study, we found no effects of ALT‐711 treatment on either aortic PWV or carotid artery passivity stiffness. We used C57BL/6 mice for this study, as they exhibit age‐dependent increases in large artery stiffness similar to those observed in humans (Longtine et al., 2023). Thus, there is potential that ALT‐711 treatment is primarily effective in the context of a disease.

Prior studies demonstrated that ALT‐711 breaks collagen crosslinks in vitro (Vasan et al., 1996). After finding no differences in large artery stiffness in these mice, we investigated the efficacy of the ALT‐711 compound in breaking crosslinks in large arteries in vivo. Increases in collagen crosslinking in the aorta with age have been well documented and align with the current understandings of vascular aging (Wang et al., 2023). We found that although old mice showed a notable increase in collagen crosslinking compared with young mice, there was no difference in crosslinking between the aortas of ALT‐711‐treated and control mice. Very few previous studies that used ALT‐711 measured collagen crosslinking (Susic et al., 2004); thus, we cannot confirm whether our findings align with the literature. Considering there were no differences in insoluble collagen content, PWV, or carotid β‐stiffness in our study, we have concluded that long‐term treatment of ALT‐711 was unsuccessful in breaking collagen crosslinking and reducing large artery stiffness.

### 4.2 Cerebrovascular function

Previous studies demonstrate that increased stiffness of the large arteries leads to impaired cerebrovascular function (Reeve et al., 2024; Tarumi et al., 2011; Walker et al., 2015). Thus, we hypothesized that reducing large artery stiffness would mitigate arterial stiffness and preserve endothelial function in cerebral arteries. However, we found that long‐term treatment with ALT‐711 did not affect cerebral artery stiffness or endothelial function, likely because it did not alter large artery stiffness. ALT‐711 could directly break crosslinks in cerebral arteries, but we were unable to assess this due to the limited tissue available.

### 4.3 Correlations between arterial stiffness and function

We combined data from ALT‐711‐treated and old control mice to determine correlations in our dataset. We found that the hydroxyproline aortic PIF: PSF, a ratio of crosslinked and non‐crosslinked collagen, was positively correlated with carotid artery stiffness measurements. This correlation further suggests that collagen crosslinking contributes to large artery stiffness, corroborating findings from many previous studies (Aronson, 2003; Hayashi & Hirayama, 2017; Reddy, 2004).

Our findings suggest that arterial stiffness adversely affects cerebral artery endothelial function and cognitive function. However, in our study, aortic PWV and carotid passive stiffness were not significantly correlated with cognitive function or cerebral artery endothelial function in our data set. Thus, it appears that cerebral artery stiffness had a greater influence on cognitive impairment and endothelial function than large elastic artery stiffness in this study. More aging research using animal models is needed to determine whether these findings are coincidental or generalizable to human aging. Increases in cerebral artery stiffness could be mediated by a remodeling response unrelated to large elastic artery stiffness, such as increased low‐grade inflammation, oxidative stress, or hypertension (Muela et al., 2017; Yang et al., 2024). We previously demonstrated that cerebral artery passive stiffness is greater with advancing age (Choi et al., 2025). These increases in cerebral artery stiffness could impact cognitive function via microvascular damage from undamped pulse pressure or impaired glymphatic clearance from reduced vascular distension (Kress et al., 2014). As it is technically difficult to measure cerebral artery stiffness in human subjects, it is unknown whether these findings translate to older human populations.

Lastly, we report that cerebral artery endothelial function was negatively correlated with frailty index scores. The frailty index score is a composite measure of biological age that incorporates multiple indicators of whole‐body health (Schultz et al., 2020; Whitehead et al., 2014). We previously found that the frailty index negatively correlated with cerebral endothelial function in old female mice, but not in old male mice (Cole et al., 2022). For the present study, we did not have large enough sample sizes to calculate correlations for each sex. Cerebral artery function may, in turn, influence whole‐body aging by regulating blood flow to key brain regions. Alternatively, frailty is a marker of biological aging, and the processes that drive aging in major organs may similarly affect the cerebral vasculature. Indeed, we previously found that the frailty index is related to resistance artery pro‐inflammatory gene expression (positively) and antioxidant gene expression (negatively) (Cole et al., 2022), suggesting a close association between “biological age” and “vascular age.”

## 5 LIMITATIONS

A limitation of this study is that animals were bred and housed in the National Institute on Aging colony at Charles River until our laboratory received them at 20 months of age. Thus, differences in living conditions between the two facilities may have affected their response to treatment. Previous studies suggest that by 20 months of age, large arteries have already stiffened in mice (DuPont et al., 2021). It is possible that the detrimental effects of arterial stiffening on cerebrovascular function and cognition were irreversible at that time. Due to the small cohort size, we were unable to compare results or determine sex differences in intervention outcomes statistically. Therefore, a future study would benefit from considering sex differences in outcomes related to large artery stiffness reduction and its effects on cerebrovascular function. Pulse pressure is an important factor that both influences and is influenced by large artery stiffness. Due to the limited cohort size, we were unable to measure pulse pressure, as this would require an additional set of mice to determine it alone. We cannot fully rule out the role that pulse pressure plays in ALT‐711's ability to reverse large artery stiffness. Therefore, it is inherently possible that ALT‐711 directly affected pulse pressure or that pulse pressure interfered with the efficacy of ALT‐711 on reducing artery stiffness.

Additionally, daily oral gavage adds additional stress to animals, and it is unclear how this stress affects the response to ALT‐711. It is possible, albeit unlikely, that the stress induced by the treatment modality minimized the beneficial effects of ALT‐711 on our measured outcomes. In this study, we did not measure ALT‐711 concentrations in the circulation or tissues of these mice. Thus, we cannot definitively determine ALT‐711 levels in our tissues of interest. The administered dose may have been too low to produce a beneficial effect on our tissues of interest, but we believe our chosen dosage level was well‐informed by previous literature (Susic et al., 2004).

In our collagen cross‐linking assessments, the young control cohort included only tissue collected from male mice, which may have inadvertently skewed the results compared with our older, mixed‐sex cohorts. We hypothesize that the collagen crosslinking results we see in our study would also apply to females. Lastly, our measurement of collagen crosslinking in this study is common but not the most accurate for determining whether crosslinks were truly broken by ALT‐711 treatment. Methods for determining collagen crosslink breakage were beyond the scope of this study, and our technique aligns with those of others in this research area (Hinks et al., 2022; Pechánová et al., 1999). Yet, whether ALT‐711 consistently breaks aortic crosslinks in vivo remains unclear, despite some evidence (Vasan et al., 1996).

## 6 CONCLUSION

In sum, ALT‐711 did not demonstrate therapeutic effects on large elastic arteries, cerebral arteries, or cognitive function in our study. These findings may suggest that ALT‐711 has greater benefits in disease conditions than in healthy aging. Interestingly, we find correlations among cerebral artery stiffness, endothelial function, and cognitive function. Future research is needed to determine the mechanisms by which cerebral artery stiffness impacts vascular and cognitive function. Identifying pharmacological approaches to ameliorate age‐related increases in cerebral arterial stiffness could help prevent cerebrovascular and cognitive decline.

## AUTHOR CONTRIBUTIONS

**Emily H. Reeve:** Conceptualization; data curation; formal analysis; funding acquisition; investigation; project administration; supervision; validation; visualization. **Abigail E. Cullen:** Data curation; investigation; methodology; validation. **Skylyn J. Ferguson:** Data curation; investigation; methodology. **Maxwell Braker:** Data curation; formal analysis; investigation; methodology. **Ainsley P. Hogan:** Data curation; formal analysis; investigation; methodology. **Alexandra Famiano:** Data curation; formal analysis; investigation; methodology. **Ashley E. Walker:** Conceptualization; data curation; formal analysis; funding acquisition; investigation; methodology; project administration; resources; supervision; validation; visualization.

## FUNDING INFORMATION

This research was supported by NIH R01 AG064016 and TL1TR002371.

## CONFLICTS OF INTEREST STATEMENT

The authors have no conflicts of interest to disclose.

## ETHICS STATEMENT

All animal procedures conform to the *Guide to the Care and Use of Laboratory Animals* (8th edition, revised 2011) and were approved by the Institutional Animal Care and Use Committees at the University of Oregon (protocol #AUP‐20‐25).

## Supporting information


**Figure S1:** Pulse wave velocity in control and ALT‐711 treated mice. (a) Pulse Wave Velocity (PWV) in control and ALT‐711 treated mice during each month of the intervention from baseline to Month 4 of the intervention, *N* = 7–8/group. Data are Mean ± SD.

**Table S1:** Animal characteristics.
**Table S2:** Heart Rate changes throughout the 4‐month intervention.

## Data Availability

Source data is available from the authors upon request.

## References

[phy270982-bib-0001] Abbott, C. B. , Lawrence, M. M. , Kobak, K. A. , Lopes, E. B. P. , Peelor, F. F., III , Donald, E. J. , Van Remmen, H. , Griffin, T. M. , & Miller, B. F. (2021). A novel stable isotope approach demonstrates surprising degree of age‐related decline in skeletal muscle collagen Proteostasis. Function, 2, zqab028. 10.1093/function/zqab028 34124684 PMC8187230

[phy270982-bib-0002] Aronson, D. (2003). Cross‐linking of glycated collagen in the pathogenesis of arterial and myocardial stiffening of aging and diabetes. Journal of Hypertension, 21, 3–12. 10.1097/00004872-200301000-00002 12544424

[phy270982-bib-0003] Baumbach, G. L. , & Hajdu, M. A. (1993). Mechanics and composition of cerebral arterioles in renal and spontaneously hypertensive rats. Hypertension, 21, 816–826. 10.1161/01.hyp.21.6.816 8500863

[phy270982-bib-0004] Chirinos, J. A. , Segers, P. , Hughes, T. , & Townsend, R. (2019). Large‐artery stiffness in health and disease: JACC state‐of‐the‐art review. Journal of the American College of Cardiology, 74, 1237–1263. 10.1016/j.jacc.2019.07.012 31466622 PMC6719727

[phy270982-bib-0005] Choi, Y. D. , Kapadia, P. , Spiegel, J. , Cullen, A. E. , LaFarga, J. , & Walker, A. E. (2025). The impact of age and sex on cerebral and large artery stiffness and the response to pulse pressure. American Journal of Physiology. Heart and Circulatory Physiology, 329, H1594–H1607. 10.1152/ajpheart.00525.2025 41187970 PMC12700119

[phy270982-bib-0006] Cole, J. A. , Kehmeier, M. N. , Bedell, B. R. , Krishna Kumaran, S. , Henson, G. D. , & Walker, A. E. (2022). Sex differences in the relation between frailty and endothelial dysfunction in old mice. The Journals of Gerontology. Series A, Biological Sciences and Medical Sciences, 77, 416–423. 10.1093/gerona/glab317 34664649 PMC8893262

[phy270982-bib-0007] Cooper, L. L. , & Mitchell, G. F. (2016). Aortic stiffness, cerebrovascular dysfunction, and memory. Pulse (Basel), 4, 69–77. 10.1159/000448176 27752478 PMC5052693

[phy270982-bib-0008] Deacon, R. (2012). Assessing burrowing, Nest construction, and hoarding in mice. Journal of Visualized Experiments, 59, e2607. 10.3791/2607 PMC3369766 22258546

[phy270982-bib-0009] Deacon, R. M. J. (2013). Measuring motor coordination in mice. Journal of Visualized Experiments, 75, e2609. 10.3791/2609 PMC3724562 23748408

[phy270982-bib-0010] Donato, A. J. , Walker, A. E. , Magerko, K. A. , Bramwell, R. C. , Black, A. D. , Henson, G. D. , Lawson, B. R. , Lesniewski, L. A. , & Seals, D. R. (2013). Life‐long caloric restriction reduces oxidative stress and preserves nitric oxide bioavailability and function in arteries of old mice. Aging Cell, 12, 772–783. 10.1111/acel.12103 23714110 PMC3772986

[phy270982-bib-0011] Dunn, W. R. , & Gardiner, S. M. (1997). Differential alteration in vascular structure of resistance arteries isolated from the cerebral and mesenteric vascular beds of transgenic [(mRen‐2)27], hypertensive rats. Hypertension, 29, 1140–1147. 10.1161/01.hyp.29.5.1140 9149679

[phy270982-bib-0012] DuPont, J. J. , Kim, S. K. , Kenney, R. M. , & Jaffe, I. Z. (2021). Sex differences in the time course and mechanisms of vascular and cardiac aging in mice: Role of the smooth muscle cell mineralocorticoid receptor. American Journal of Physiology. Heart and Circulatory Physiology, 320, H169–H180. 10.1152/ajpheart.00262.2020 33095647 PMC7847078

[phy270982-bib-0013] Duvoisin, R. M. , Villasana, L. , Pfankuch, T. , & Raber, J. (2010). Sex‐dependent cognitive phenotype of mice lacking mGluR8. Behavioural Brain Research, 209, 21–26. 10.1016/j.bbr.2010.01.006 20080129 PMC2832071

[phy270982-bib-0014] Faakye, A. , Harold, K. M. , Matsuzaki, S. , Pranay, A. , Mendez Garcia, M. F. , Loveland, B. L. , Rigsby, S. N. , Peelor, F. F. , Eyster, C. , Miller, B. F. , Griffin, T. M. , Kinter, M. , Chiao, Y. A. , & Humphries, K. M. The Effect of Enhanced Glycolysis on Cardiac Aging. 10.1007/s11357-025-01656-z PMC12634992 40310487

[phy270982-bib-0015] Freidja, M. L. , Tarhouni, K. , Toutain, B. , Fassot, C. , Loufrani, L. , & Henrion, D. (2012). The AGE‐breaker ALT‐711 restores high blood flow–dependent remodeling in mesenteric resistance arteries in a rat model of type 2 diabetes. Diabetes, 61, 1562–1572. 10.2337/db11-0750 22415880 PMC3357287

[phy270982-bib-0016] Hartog, J. W. L. , Willemsen, S. , van Veldhuisen, D. J. , Posma, J. L. , van Wijk, L. M. , Hummel, Y. M. , Hillege, H. L. , Voors, A. A. , & BENEFICIAL investigators . (2011). Effects of alagebrium, an advanced glycation endproduct breaker, on exercise tolerance and cardiac function in patients with chronic heart failure. European Journal of Heart Failure, 13, 899–908. 10.1093/eurjhf/hfr067 21669961

[phy270982-bib-0017] Hayashi, K. , & Hirayama, E. (2017). Age‐related changes of wall composition and collagen cross‐linking in the rat carotid artery ‐ in relation with arterial mechanics. Journal of the Mechanical Behavior of Biomedical Materials, 65, 881–889. 10.1016/j.jmbbm.2016.10.007 27821371

[phy270982-bib-0018] Henson, G. D. , Walker, A. E. , Reihl, K. D. , Donato, A. J. , & Lesniewski, L. A. (2014). Dichotomous mechanisms of aortic stiffening in high‐fat diet fed young and old B6D2F1 mice. Physiological Reports, 2, e00268. 10.1002/phy2.268 24760522 PMC4002248

[phy270982-bib-0019] Hinks, A. , Jacob, K. , Mashouri, P. , Medak, K. D. , Franchi, M. V. , Wright, D. C. , Brown, S. H. M. , & Power, G. A. (2022). Influence of weighted downhill running training on serial sarcomere number and work loop performance in the rat soleus. Biol Open, 11, bio059491. 10.1242/bio.059491 35876382 PMC9346294

[phy270982-bib-0020] Izzard, A. S. , Horton, S. , Heerkens, E. H. , Shaw, L. , & Heagerty, A. M. (2006). Middle cerebral artery structure and distensibility during developing and established phases of hypertension in the spontaneously hypertensive rat. Journal of Hypertension, 24, 875–880. 10.1097/01.hjh.0000222757.54111.06 16612249

[phy270982-bib-0021] Kass, D. A. , Shapiro, E. P. , Kawaguchi, M. , Capriotti, A. R. , Scuteri, A. , deGroof, R. C. , & Lakatta, E. G. (2001). Improved arterial compliance by a novel advanced glycation end‐product crosslink breaker. Circulation, 104, 1464–1470. 10.1161/hc3801.097806 11571237

[phy270982-bib-0022] Kress, B. T. , Iliff, J. J. , Xia, M. , Wang, M. , Wei, H. S. , Zeppenfeld, D. , Xie, L. , Kang, H. , Xu, Q. , Liew, J. A. , Plog, B. A. , Ding, F. , Deane, R. , & Nedergaard, M. (2014). Impairment of paravascular clearance pathways in the aging brain. Annals of Neurology, 76, 845–861. 10.1002/ana.24271 25204284 PMC4245362

[phy270982-bib-0023] Lesniewski, L. A. , Connell, M. L. , Durrant, J. R. , Folian, B. J. , Anderson, M. C. , Donato, A. J. , & Seals, D. R. (2009). B6D2F1 mice are a suitable model of oxidative stress‐mediated impaired endothelium‐dependent dilation with aging. The Journals of Gerontology. Series A, Biological Sciences and Medical Sciences, 64, 9–20. 10.1093/gerona/gln049 19211548 PMC2691198

[phy270982-bib-0024] Longtine, A. G. , Venkatasubramanian, R. , Zigler, M. C. , Lindquist, A. J. , Mahoney, S. A. , Greenberg, N. T. , VanDongen, N. S. , Ludwig, K. R. , Moreau, K. L. , Seals, D. R. , & Clayton, Z. S. (2023). Female C57BL/6N mice are a viable model of aortic aging in women. American Journal of Physiology. Heart and Circulatory Physiology, 324, H893–H904. 10.1152/ajpheart.00120.2023 37115626 PMC10202480

[phy270982-bib-0025] Luevano‐Contreras, C. , & Chapman‐Novakofski, K. (2010). Dietary advanced glycation end products and aging. Nutrients, 2, 1247–1265. 10.3390/nu2121247 22254007 PMC3257625

[phy270982-bib-0026] Mammoto, A. , Matus, K. , & Mammoto, T. (2022). Extracellular matrix in aging aorta. Frontiers in Cell and Development Biology, 10, 822561. 10.3389/fcell.2022.822561 PMC8898904 35265616

[phy270982-bib-0027] Mitchell, G. F. , Parise, H. , Benjamin, E. J. , Larson, M. G. , Keyes, M. J. , Vita, J. A. , Vasan, R. S. , & Levy, D. (2004). Changes in arterial stiffness and wave reflection with advancing age in healthy men and women: The Framingham heart study. Hypertension, 43, 1239–1245. 10.1161/01.HYP.0000128420.01881.aa 15123572

[phy270982-bib-0028] Muela, H. C. S. , Costa‐Hong, V. A. , Yassuda, M. S. , Machado, M. F. , de Nogueira, R. C. , Moraes, N. C. , Memória, C. M. , Macedo, T. A. , Bor‐Seng‐Shu, E. , Massaro, A. R. , Nitrini, R. , & Bortolotto, L. A. (2017). Impact of hypertension severity on arterial stiffness, cerebral vasoreactivity, and cognitive performance. Dement Neuropsychol, 11, 389–397. 10.1590/1980-57642016dn11-040008 29354219 PMC5769997

[phy270982-bib-0029] Pechánová, O. , Bernátová, I. , Pelouch, V. , & Babál, P. (1999). L‐NAME‐induced protein remodeling and fibrosis in the rat heart. Physiological Research, 48, 353–362. 10625224

[phy270982-bib-0030] Peppa, M. , Uribarri, J. , & Vlassara, H. (2008). Aging and glycoxidant stress. Hormones (Athens, Greece), 7, 123–132. 10.1007/BF03401503 18477549

[phy270982-bib-0031] Rabkin, S. W. (2012). Arterial stiffness: Detection and consequences in cognitive impairment and dementia of the elderly. Journal of Alzheimer's Disease, 32, 541–549. 10.3233/JAD-2012-120757 22886012

[phy270982-bib-0032] Reddy, G. K. (2004). AGE‐related cross‐linking of collagen is associated with aortic wall matrix stiffness in the pathogenesis of drug‐induced diabetes in rats. Microvascular Research, 68, 132–142. 10.1016/j.mvr.2004.04.002 15313123

[phy270982-bib-0033] Reeve, E. H. , Barnes, J. N. , Moir, M. E. , & Walker, A. E. (2024). Impact of arterial stiffness on cerebrovascular function: A review of evidence from humans and preclincal models. American Journal of Physiology. Heart and Circulatory Physiology, 326, H689–H704. 10.1152/ajpheart.00592.2023 38214904 PMC11221809

[phy270982-bib-0034] Reeve, E. H. , Kronquist, E. K. , Wolf, J. R. , Lee, B. , Khurana, A. , Pham, H. , Cullen, A. E. , Peterson, J. A. , Meza, A. , Colton Bramwell, R. , Villasana, L. , Machin, D. R. , Henson, G. D. , & Walker, A. E. (2023). Pyridoxamine treatment ameliorates large artery stiffening and cerebral artery endothelial dysfunction in old mice. Journal of Cerebral Blood Flow and Metabolism, 43, 281–295. 10.1177/0271678X221130124 36189840 PMC9903220

[phy270982-bib-0035] Ruitenberg, A. , den Heijer, T. , Bakker, S. L. M. , van Swieten, J. C. , Koudstaal, P. J. , Hofman, A. , & Breteler, M. M. B. (2005). Cerebral hypoperfusion and clinical onset of dementia: The Rotterdam study. Annals of Neurology, 57, 789–794. 10.1002/ana.20493 15929050

[phy270982-bib-0036] Sajithlal, G. B. , Chithra, P. , & Chandrakasan, G. (1998). Advanced glycation end products induce crosslinking of collagen in vitro. Biochimica et Biophysica Acta, 1407, 215–224. 10.1016/s0925-4439(98)00043-x 9748585

[phy270982-bib-0037] Schultz, M. B. , Kane, A. E. , Mitchell, S. J. , MacArthur, M. R. , Warner, E. , Vogel, D. S. , Mitchell, J. R. , Howlett, S. E. , Bonkowski, M. S. , & Sinclair, D. A. (2020). Age and life expectancy clocks based on machine learning analysis of mouse frailty. Nature Communications, 11, 4618. 10.1038/s41467-020-18446-0 PMC7492249 32934233

[phy270982-bib-0038] Sell, D. R. , & Monnier, V. M. (2012). Molecular basis of arterial stiffening: Role of glycation ‐ a mini‐review. Gerontology, 58, 227–237. 10.1159/000334668 22222677

[phy270982-bib-0039] Semba, R. D. , Bandinelli, S. , Sun, K. , Guralnik, J. M. , & Ferrucci, L. (2009). Plasma carboxymethyl‐lysine, an advanced glycation end product, and all‐cause and cardiovascular disease mortality in older community‐dwelling adults. Journal of the American Geriatrics Society, 57, 1874–1880. 10.1111/j.1532-5415.2009.02438.x 19682127 PMC2785105

[phy270982-bib-0040] Smith, L. R. , Hammers, D. W. , Sweeney, H. L. , & Barton, E. R. (2016). Increased collagen cross‐linking is a signature of dystrophin‐deficient muscle. Muscle & Nerve, 54, 71–78. 10.1002/mus.24998 26616495 PMC5067682

[phy270982-bib-0041] Stone, J. , Johnstone, D. M. , Mitrofanis, J. , & O'Rourke, M. (2015). The mechanical cause of age‐related dementia (Alzheimer's disease): The brain is destroyed by the pulse. Journal of Alzheimer's Disease, 44, 355–373. 10.3233/JAD-141884 25318547

[phy270982-bib-0042] Susic, D. , Varagic, J. , Ahn, J. , & Frohlich, E. D. (2004). Cardiovascular and renal effects of a collagen cross‐link breaker (ALT 711) in adult and aged spontaneously hypertensive rats. American Journal of Hypertension, 17, 328–333. 10.1016/j.amjhyper.2003.12.015 15062886

[phy270982-bib-0043] Tarumi, T. , Shah, F. , Tanaka, H. , & Haley, A. P. (2011). Association between central elastic artery stiffness and cerebral perfusion in deep subcortical gray and white matter. American Journal of Hypertension, 24, 1108–1113. 10.1038/ajh.2011.101 21654859

[phy270982-bib-0044] Thorin‐Trescases, N. , de Montgolfier, O. , Pinçon, A. , Raignault, A. , Caland, L. , Labbé, P. , & Thorin, E. (2018). Impact of pulse pressure on cerebrovascular events leading to age‐related cognitive decline. American Journal of Physiology. Heart and Circulatory Physiology, 314, H1214–H1224. 10.1152/ajpheart.00637.2017 29451817 PMC6032083

[phy270982-bib-0045] Toprak, C. , Sirmagul, B. , & Yigitaslan, S. (2017). Functional effects of Alagebrium (ALT‐711)–isolated rat carotid artery. Eurasian J Med, 49, 188–192. 10.5152/eurasianjmed.2017.17046 29123442 PMC5665628

[phy270982-bib-0046] Vaitkevicius, P. V. , Lane, M. , Spurgeon, H. , Ingram, D. K. , Roth, G. S. , Egan, J. J. , Vasan, S. , Wagle, D. R. , Ulrich, P. , Brines, M. , Wuerth, J. P. , Cerami, A. , & Lakatta, E. G. (2001). A cross‐link breaker has sustained effects on arterial and ventricular properties in older rhesus monkeys. Proceedings of the National Academy of Sciences of the United States of America, 98, 1171–1175. 10.1073/pnas.98.3.1171 11158613 PMC14727

[phy270982-bib-0047] van Sloten, T. T. , Protogerou, A. D. , Henry, R. M. A. , Schram, M. T. , Launer, L. J. , & Stehouwer, C. D. A. (2015). Association between arterial stiffness, cerebral small vessel disease and cognitive impairment: A systematic review and meta‐analysis. Neuroscience and Biobehavioral Reviews, 53, 121–130. 10.1016/j.neubiorev.2015.03.011 25827412 PMC5314721

[phy270982-bib-0048] Vasan, S. , Zhang, X. , Zhang, X. , Kapurniotu, A. , Bernhagen, J. , Teichberg, S. , Basgen, J. , Wagle, D. , Shih, D. , Terlecky, I. , Bucala, R. , Cerami, A. , Egan, J. , & Ulrich, P. (1996). An agent cleaving glucose‐derived protein crosslinks in vitro and in vivo. Nature, 382, 275–278. 10.1038/382275a0 8717046

[phy270982-bib-0049] Walker, A. E. , Henson, G. D. , Reihl, K. D. , Morgan, R. G. , Dobson, P. S. , Nielson, E. I. , Ling, J. , Mecham, R. P. , Li, D. Y. , Lesniewski, L. A. , & Donato, A. J. (2015). Greater impairments in cerebral artery compared with skeletal muscle feed artery endothelial function in a mouse model of increased large artery stiffness. The Journal of Physiology, 593, 1931–1943. 10.1113/jphysiol.2014.285338 25627876 PMC4405752

[phy270982-bib-0050] Wang, H. , Weihrauch, D. , Kersten, J. R. , Toth, J. M. , Passerini, A. G. , Rajamani, A. , Schrepfer, S. , & LaDisa, J. F., Jr. (2015). Alagebrium inhibits neointimal hyperplasia and restores distributions of wall shear stress by reducing downstream vascular resistance in obese and diabetic rats. American Journal of Physiology Heart and Circulatory Physiology, 309, H1130–H1140. 10.1152/ajpheart.00123.2014 26254329 PMC4865061

[phy270982-bib-0051] Wang, R. , Mattson, J. M. , & Zhang, Y. (2023). Effect of aging on the biaxial mechanical behavior of human descending thoracic aorta: Experiments and constitutive modeling considering collagen crosslinking. Journal of the Mechanical Behavior of Biomedical Materials, 140, 105705. 10.1016/j.jmbbm.2023.105705 36758423 PMC10023391

[phy270982-bib-0052] Whitehead, J. C. , Hildebrand, B. A. , Sun, M. , Rockwood, M. R. , Rose, R. A. , Rockwood, K. , & Howlett, S. E. (2014). A clinical frailty index in aging mice: Comparisons with frailty index data in humans. The Journals of Gerontology. Series A, Biological Sciences and Medical Sciences, 69, 621–632. 10.1093/gerona/glt136 24051346 PMC4022099

[phy270982-bib-0053] Wolffenbuttel, B. H. , Boulanger, C. M. , Crijns, F. R. , Huijberts, M. S. , Poitevin, P. , Swennen, G. N. , Vasan, S. , Egan, J. J. , Ulrich, P. , Cerami, A. , & Lévy, B. I. (1998). Breakers of advanced glycation end products restore large artery properties in experimental diabetes. Proceedings of the National Academy of Sciences of the United States of America, 95, 4630–4634. 10.1073/pnas.95.8.4630 9539789 PMC22541

[phy270982-bib-0054] Yang, D. , Cherian, L. , Arfanakis, K. , Schneider, J. A. , Aggarwal, N. T. , & Gutierrez, J. (2024). Intracranial atherosclerotic disease and neurodegeneration: A narrative review and plausible mechanisms. Journal of Stroke and Cerebrovascular Diseases: The Official Journal of National Stroke Association, 33, 108015. 10.1016/j.jstrokecerebrovasdis.2024.108015 39303868 PMC11570339

