# Supplementary figures and images for: Age‐related arterial stiffness and cerebrovascular dysfunction in mice correlate with cognitive impairment but are not reduced with long‐term ALT‐711 treatment
Source: Physiol Rep. 2026 Jun 17;14(12):e70982. doi: 10.14814/phy2.70982 (PMC13276286; doi:10.14814/phy2.70982)

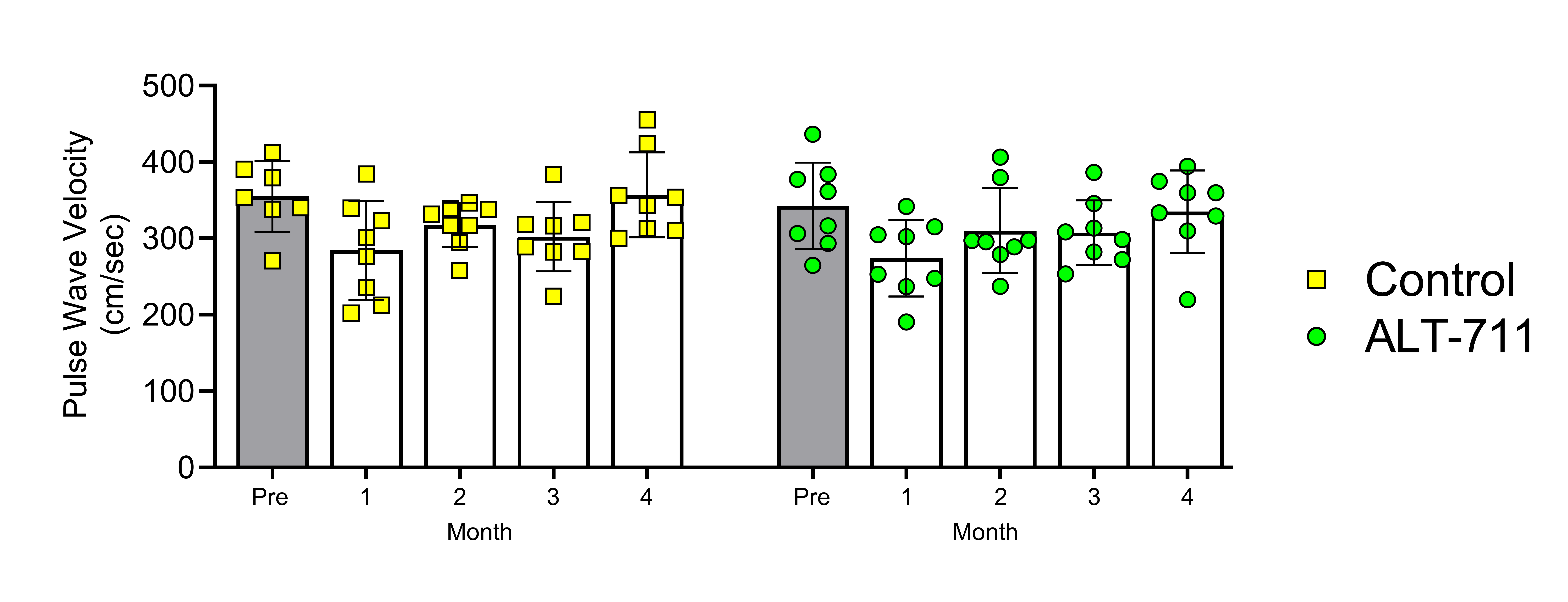

Supplement: Supplementary file 1 — Figure S1: Pulse wave velocity in control and ALT‐711 treated mice. (a) Pulse Wave Velocity (PWV) in control and ALT‐711 treated mice during each month of the intervention from baseline to Month 4 of the intervention, N = 7–8/group. Data are Mean ± SD. [file PHY2-14-e70982-s001.tif]
