# Supplementary material for: Age‐related arterial stiffness and cerebrovascular dysfunction in mice correlate with cognitive impairment but are not reduced with long‐term ALT‐711 treatment
Source: Physiol Rep. 2026 Jun 17;14(12):e70982. doi: 10.14814/phy2.70982 (PMC13276286; doi:10.14814/phy2.70982)
Supplement: Supplementary file 2 — Table S1: Animal characteristics. Table S2: Heart Rate changes throughout the 4‐month intervention. [file PHY2-14-e70982-s002.docx]

**Supplemental Table 1**. Animal Characteristics

| Variable | Control | ALT-711 |
| --- | --- | --- |
| N | 8 (4 F, 4 M) | 8 (4 F, 4 M) |
| age (months) | 23.9 ± 0.1 | 23.9 ± 0.1 |
| Body mass (g) | 30.5 ± 0.7 | 30.0 ± 2.8 |
| Heart mass, mg | 153 ± 11 | 149 ± 29 |
| Percent heart: body mass | 0.52 ± 0.05 | 0.47 ± 0.05 |
| Liver mass, mg | 1646 ± 155 | 1507 ± 332 |
| Percent liver: body mass | 5.5 ± 0.26 | 5.0 ± 0.69 |
| Spleen mass, mg | 105 ± 33 | 120 ± 35 |
| Percent spleen: body mass | 0.34 ± 0.11 | 0.41 ± 0.14 |
| WAT mass, mg | 555 ± 227 | 789 ± 469 |
| Percent WAT: body mass | 1.8 ± 0.70 | 2.6 ± 1.5 |
| Gastroc mass, mg | 123 ± 23 | 116 ± 10 |
| Percent gastroc: body mass | 0.42 ± 0.07 | 0.39 ± 0.05 |
| Soleus mass, mg | 15 ± 7 | 12 ± 4 |
| Percent soleus: body mass | 0.04 ± 0.01 | 0.03 ± 0.02 |
| Kidneys mass, mg | 525 ± 44 | 487 ± 57 |
| Percent kidneys: body mass | 1.7 ± 0.19 | 1.6 ± 0.17 |
| Frailty Index | 0.26 ± 0.03 | 0.27 ± 0.05 |

**Supplemental Table 1. Animal Characteristics.** Age, body mass, tissue weights, and frailty index for animals that were included in this study. Data are Mean±SD. White adipose tissue (WAT), gastrocnemius (gastroc).

**Supplemental Table 2. Heart Rate changes throughout the 4-month intervention**

|  | Heart Rate (bpm) | |
| --- | --- | --- |
| Group | Control | ALT-711 |
|  |  |  |
| Baseline | 470±25 | 463±26 |
| Month 1 | 471±56 | 481±35 |
| Month 2 | 467±46 | 460±46 |
| Month 3 | 506±43 | 502±32 |
| Month 4 | 498±37 | 482±43 |

**Supplemental Table 2. Average heart rate is unchanged and not different between groups throughout the 4-month intervention.** Heart rate was calculated from pulse wave velocity data. Data are Mean±SD.
